# Analysis of Experimental Biaxial Surface Wrinkling Pattern Based on Direct 3D Numerical Simulation

**DOI:** 10.3390/mi15040543

**Published:** 2024-04-18

**Authors:** Seonho Seok, HyungDal Park, Jinseok Kim

**Affiliations:** 1 Center for Nanoscience and Nanotechnology (C2N), Université Paris-Saclay, 91129 Palaiseau, France; seonho.seok@c2n.upsaclay.fr; 2 Center for Bionics, Korea Institute of Science and Technology, Seoul 02792, Republic of Korea; hyungdal@kist.re.kr

**Keywords:** biaxial wrinkling, buckling, fem, direct simulation, 3D, transfer

## Abstract

This paper presents a direct 3D numerical simulation of biaxial surface wrinkling of thin metal film on a compliant substrate. The selected compliant substrate is a commercial Scotch tape on which a gold metal thin film has been transferred by using low adhesion between the thin metal film and polyimide substrate. Compared with the previous fabrication of a cylindrical thin-film wrinkling pattern, an undulated wrinkling pattern has been implemented by increasing the width of the thin metal film in order to create biaxial straining in the thin film. To understand the wrinkling behavior due to biaxial loading, a simple direct numerical simulation based on material imperfections defined in the compliant substrate has been conducted. Through modeling and simulation, it was found that the wrinkling mode is determined by the biaxiality ratio (BR), the ratio between transversal strain and longitudinal strain. Depending on the BR, the wrinkling mode belongs to one of the cylindrical, undulated (or herringbone), checkerboard, or labyrinth modes as a function of applied strain. The cylindrical wrinkling is dominant at the input of BR less than 0.5, while the undulated (or herringbone) ones become dominant just after the onset of the wrinkling pattern at BR greater than 0.9. Through the comparison of the wrinkling patterns between simulation and experiment, the applied BR of the fabricated thin film has been successfully estimated.

## 1. Introduction

Buckling of multi-layered material is a classical subject and has received continuous attention in mechanics due to its various applications, such as sandwich panels. Concerning microfabrication, such buckling has been frequently observed when thin and stiff film is boned to a compliant substrate. This can happen experimentally by depositing a thin metal film onto a compliant substrate, such as an uncured polymer substrate, and letting the system cool down to room temperature. Surface wrinkling driven by mechanical instability is commonly observed in different thin-film structures with a compliant substrate [1,2,3,4,5]. Apart from the metal deposition mentioned earlier, such surface wrinkling patterns have been engineered with different techniques such as surface oxidation of PDMS [6], thin metal film deposition on thermally expanded polymer [7], polymer mold confinement on buckling of thin film [8], etc. Significant progress in theoretical and experimental studies has led to a greater physical understanding of surface instability phenomena, spurring research interest in engineering topologically self-organizing systems across many length scales [9]. Recently, surface wrinkling based on the thin-film buckling phenomenon has been proposed to find the elastic moduli of thin films such as thin polymer films, thin film metals, carbon nanotubes, etc. [10]. This technique allows us to calculate the elastic modulus of a thin film by measuring the wavelength of the wrinkling thin film. Elastomer material such as PDMS is frequently used as a base substrate as it has a low elastic modulus. Commercial Scotch tape having adhesive on a supporting membrane such as PVC, PET, and PE was also reported as a buckling substrate with an elastic thin film [11].

For most of the reports on wrinkling studies and material properties extraction from wrinkling patterns, the analytical formulation has been widely employed in analyzing experimental data, for example, wrinkling wavelengths [12,13,14]. Additionally, numerical studies have been carried out to explore the underlying buckling mechanism as well as to quantitatively control and manipulate the self-assembled patterns for applications in micro/nanofabrication [15,16,17]. Modeling and simulation based on finite element modeling (FEM) are useful to analyze the surface wrinkling pattern depending on various geometries, different material properties, etc. The FEM models based on 2D or 3D have successfully provided wrinkling wavelengths as a function of material properties associated with surface wrinkling. As for finite element modeling associated with instabilities, it is conventional that linear buckling for wrinkling wavelength based on basic buckling mode and post-buckling for wrinkling amplitude based on buckling mode from pre-performed linear buckling analysis have been used in the studies of the surface wrinkling phenomenon [18,19,20]. These simulations have been, in general, realized by buckling analysis established in commercial software packages such as ABAQUS and ANSYS. Geometrical imperfection methods have been frequently employed by using mesh, geometry, and boundary condition perturbation techniques for studying surface instability. However, the existing imperfection approaches are relatively complicated, and the implementation can be laborious due to free parameter calibration. Furthermore, the interpretation and verification of results are not straightforward, which makes it less practical for common users. To overcome the drawbacks of the geometrical imperfection methods, a direct FEM simulation method that uses embedded imperfections with perturbed material properties at the film-substrate interface has been recently proposed. The direct simulation can successfully perform the pre/post-buckling simulations in only one analysis step with any common finite element code and analysis platform [21,22,23]. The direct FEM model can provide the wrinkling wavelength of a surface wrinkling as a function of material properties, and thus the extraction of material properties such as Young’s modulus has been achieved [24]. In this paper, a direct 3D numerical simulation of biaxial surface wrinkling of thin metal film on a compliant substrate is presented to comprehend the experimentally created wrinkling pattern. The theoretical background of thin-film wrinkling on the compliant substrate is explained in Section 2, and the fabrication process of the wrinkling pattern is depicted in Section 3. Section 4 shows the results and discussions of the FEM modeling and simulation through comparison with experimental results. Finally, the conclusion will be made in Section 5.

## 2. Brief Theoretical Description of Wrinkling of Thin Film on a Substrate 

**Figure 1:**
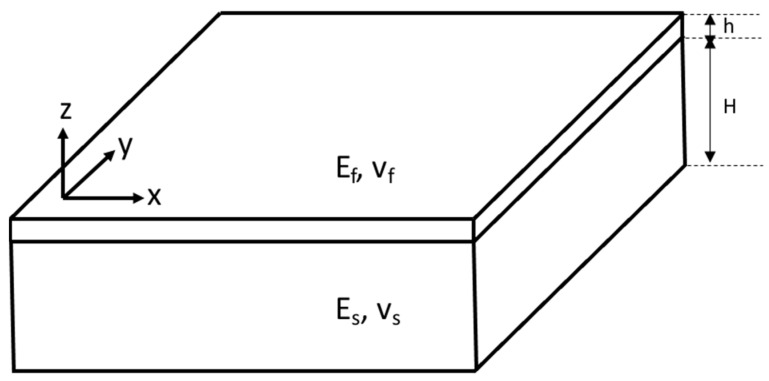
Geometrical schematics of the thin-film wrinkling problem.

The force balance approach for surface buckling instability of a thin film on a compliant substrate is here briefly introduced [10]. The wrinkling problem can be described such that a thin elastic film is bound to an elastic foundation, as shown in Figure 1. The source of the wrinkling is the compressive stress of the film.

Considered as a semi-infinite substrate under plane strain deformation, the classical equation for the bending of an elastic film on a compliant elastic substrate is given by Equation (1).

(1) $$\bar{E_{f}}I\frac{d^{4}z}{dx^{4}}+F\frac{d^{2}z}{dx^{2}}+k_{m}z=0$$

where $\bar{E}$ = *E*/(1 − *ν*^2^) is the plane-strain modulus, *E* is Young’s modulus, *v* is Poisson’s ratio, *I* = wh^3^/12 is the moment of inertia (where w is the width of the film and h is its thickness), *F* is the uniaxially applied force or load, and *k* is Winkler’s modulus of an elastic half-space (*k_m_* =$\;\bar{E_{s}}w\pi /\lambda )$. The subscripts, *f* and *s*, denote the film and substrate, respectively.

In Equation (1), the first and third terms are along with the classical Euler–Bernoulli beam-bending equation that equalizes bending forces in the film with the normal force distribution due to the deformation of the substrate above and below its neutral position. The second term means the effects of the force, *F*, on the film. In contrast to the buckling of a column, the wrinkling of a film on an elastic foundation involves a balance of force between film bending (first term in Equation (1)), which acts to suppress short wavelengths, and substrate deformation (third term in Equation (1)), which acts to suppress large wavelengths [10].

As the buckling instability of interest here is the first sinusoidal mode, the film deflection can be described by Equation (2).

(2) $$w\left(x\right)=A\cos\frac{2\pi x}{\lambda }=Acos\;kx$$

Substituting Equation (2) into Equation (1) and solving for the applied force in the thin film on a compliant substrate gives Equation (3).

(3) $$F=4\bar{E_{f}}I{\left(\frac{\pi }{\lambda }\right)}^{2}+\frac{\bar{E_{s}}w}{4}{\left(\frac{\pi }{\lambda }\right)}^{-1}$$

The film buckling wavelength can be found by minimizing *F* with respect to *λ* (or, ∂F/∂λ=0),

(4) $$\lambda =2\pi h{\left(\frac{\bar{E_{f}}}{3\bar{E_{s}}}\right)}^{\frac{1}{3}}$$

It should be noted here that the wavelength is only a function of the thickness of the film and the elastic properties of the film and substrate. Thus, the wrinkling wavelength can be used to determine the material properties of the thin film if the substrate material properties are known.

Another important parameter is the critical stress or strain needed to induce wrinkling in the system. The critical stress can be found in Equations (4) and (5) by dividing the critical force (*Fc*) by the cross-sectional area of the thin film.

(5) $$\sigma_{c}=\frac{F_{c}}{hw}={\left(\frac{9}{64}\bar{E_{f}}\;\;{\bar{E_{s}}}^{2}\right)}^{\frac{1}{3}}$$

Therefore, the critical strain is given below.

(6) $$\varepsilon_{c}=\frac{\sigma_{c}}{\bar{E_{c}}}={\frac{1}{4}\left(\frac{3\bar{E_{S}}}{\bar{E_{f}}}\;\;\right)}^{\frac{2}{3}}$$

Under the assumption that the wrinkling wavelength is independent of the applied strain, the wrinkling amplitude can be found in Equation (7).

(7) $$A=h\sqrt{\frac{\varepsilon -\varepsilon_{c}}{\varepsilon_{c}}}$$

where *A* is wrinkling amplitude, and ε is applied strain.

In the case of biaxial straining, an infinitesimal perturbation can be introduced to the deflection of the film as follows [25]:

(8) $$w\left(x,y\right)=Acos\;kx+\delta w\left(x,y\right)$$

Note that the first term is the same one in uniaxial straining, as explained above.

By applying the wrinkling behaviors in the *x* and *y* axes, the perturbation equation can be expressed as follows [25].

(9) $$\delta w\left(x,y\right)=A_{1}\cos\left(k_{x}x+k_{y}y\right)+A_{2}\cos\left(k_{x}x-k_{y}y\right)+A_{3}\cos\left(k_{y}y\right)$$

where *k_x_* and *k_y_* are the associated wavenumbers, and *A*_1_ is the arbitrary amplitude.

Using trigonometric identities, the deflection of the film, including the base solution *A*cos(*kx*), can be then written as:

(10) $$\delta w\left(x,y\right)=A\cos\left(k_{x}x\right)+b\sin\left(k_{x}x\right)\sin\left(k_{y}qy\right)+c\cos\left(k_{x}x\right)\cos\left(k_{y}qy\right)+d\;\cos(k_{y}qy)$$

where *q* = *k_y_*/*k_x_* is the rescaled longitudinal wavevector, *b* = *A*_2_ − *A*_1_, *c* = *A*_1_ + *A*_2_, and *d* = *A*_3_. It is noted that the numbers *b*, *c*, and *d* appear as amplitudes of the undulating mode, the varicose mode, and the checkerboard mode, respectively. The amplitudes *b*, *c*, and *d* are taken to be infinitesimal, and thus, linear stability is not considered in this present form.

**Figure 2:**
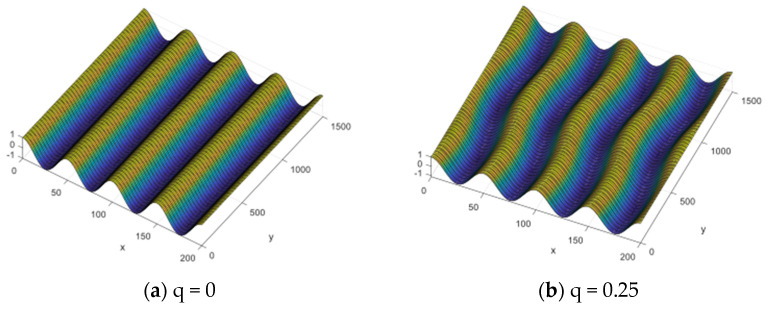

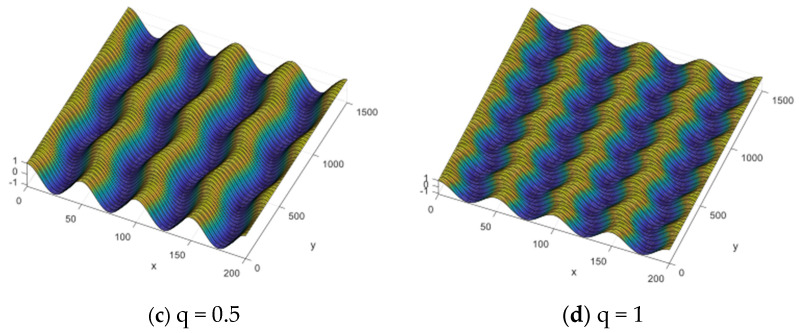
Wrinkling patterns as a function of parameter q.

Biaxial wrinkling patterns depending on parameter *q* are given in Figure 2. The wrinkling pattern has been defined as *λ_x_* = 50 μm and *λ_y_* = 250 μm. A cylindrical wrinkling pattern is found when *q* is zero, as expected. When *q* is 0.25, it is confirmed that the 4-cycle wrinkling pattern is established in the *x* direction, while the 1-cycle pattern is made in the *y* direction by definition of the parameter *q*. The other wrinkling patterns have shown the same tendency, depending on the *q*.

## 3. Fabrication of Biaxial Thin-Film Wrinkling on Scotch Tape

**Figure 3:**
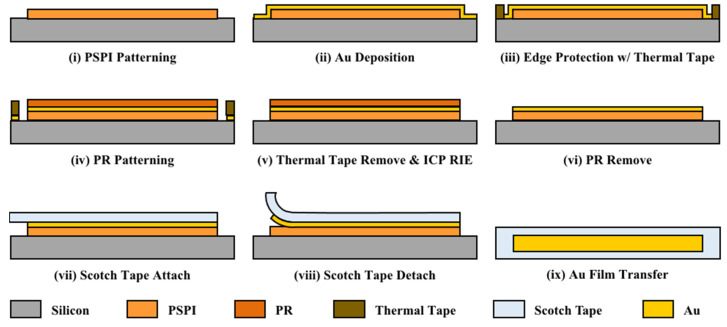
Thin-film metal test pattern fabrication process.

To make metal wrinkling patterns on Scotch tape, thin metal films have been deposited on a PSPI (Photosensitive Polyimide) layer built on Si substrate by e-beam evaporation (ei-5k, ULVAC, Chigasaki, Japan). As in the previous work, the PSPI layer has been used because it has a low adhesion force to the metal films. Figure 3 shows the fabrication process of the test sample.

First, the native oxide and organic matter formed on the Si wafer surface were removed through a general piranha cleaning solution, which is a 3:1 mixture of sulfuric acid and 30% hydrogen peroxide. Thereafter, the PSPI was coated to a thickness of 5 μm with a 4500 rpm/1 min condition using a spin coater. The coated PSPI was soft-baked at 110 °C for 6 min, and 200 mJ UV was exposed to PSPI using the aligner equipment (MA-6 Mask Aligner, Karl Suss, Munich, Germany). Afterward, PEB (Post-Exposure Baking) was performed at 110 °C for 5 min, and a pattern was developed using a dedicated developer (401D, HD MicroSystems™, Parlin, NJ, USA) and Rince (AZ Thinner 1500, MERCK, Rahway, NJ, USA). The patterned PSPI was subjected to PDB (Post-Develop Baking) at 180 °C for at least 1 min, and full curing was performed for 30 min at 200 °C and at 300 °C for 1 h in a vacuum drying oven. (Customized Oven, SH Scientific Co., Ltd., Sejong, Republic of Korea). 

**Figure 4:**
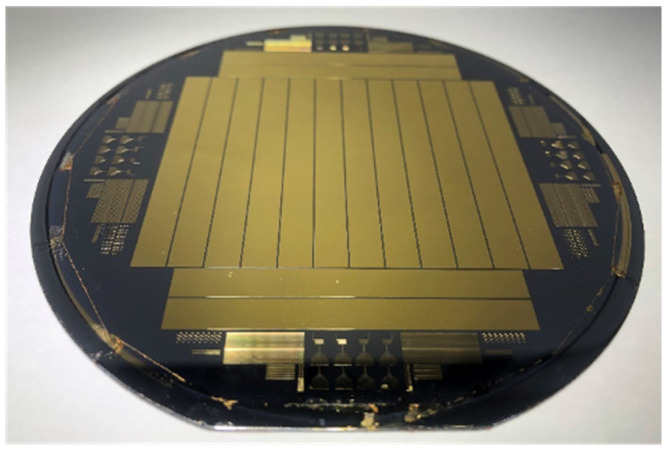
Fabrication result of the thin-film metal test pattern.

Au (gold) was deposited on a full-cured PSPI to a thickness of 300 nm using an E-beam evaporator. Subsequently, a commercial thermal adhesive film was applied to safeguard the Au boundary with the Si surface formed at the periphery of the Si wafer. Patterning was performed on the deposited metal thin film using a positive photoresist (GXR-601 46cp, AZ Electronics Materials, Hsinchu Country, Taiwan), and Au was selectively dry etched using an ICP-RIE (Inductively Coupled Plasma Reactive Ion Etcher, Oxford Instruments, Abingdon, UK). Considering the low adherence of the formed Au pattern to PSPI, the photoresist removal was completed by using O_2_ plasma at 300 W for 5 min using Asher equipment (Plasma Finish V15-G, Ebhausen, Germany). Figure 4 shows an Au thin film fabricated on the PSPI polymer with a Si substrate.

**Figure 5:**
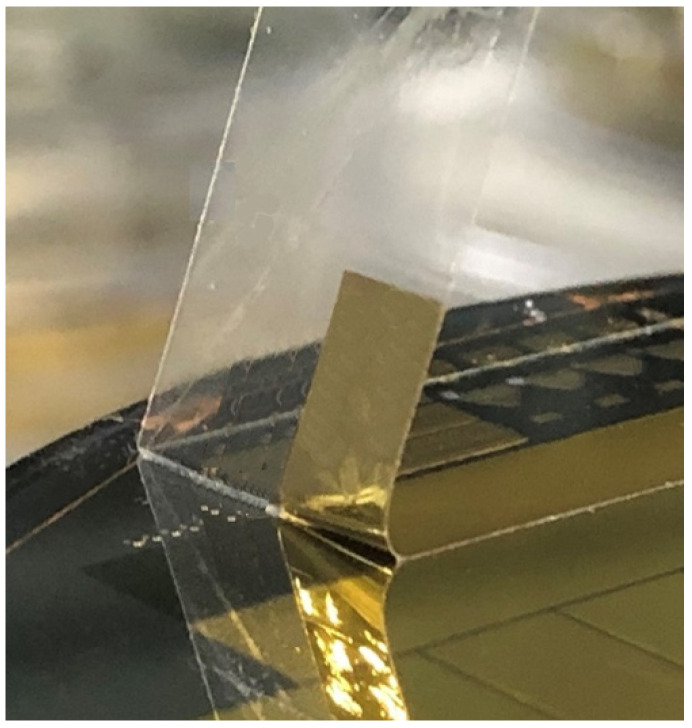
Gold thin film from PSPI substrate to Scotch tape.

**Figure 6:**
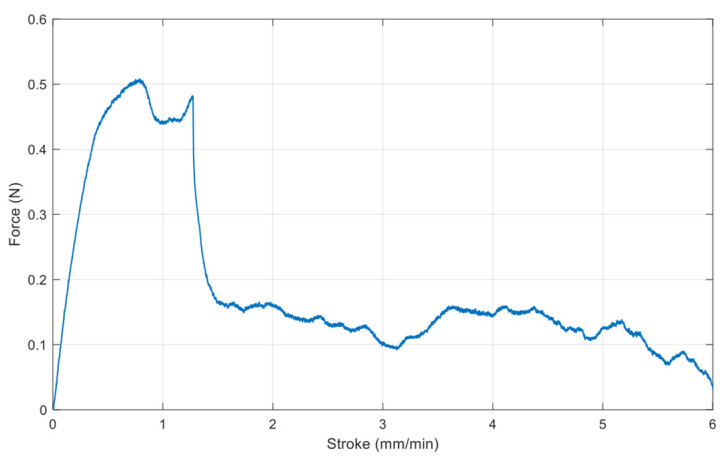
Debonding force measurement of the Scotch tape.

The gold thin film was transferred onto Scotch tape by commercial tensile experimental equipment (Shimadzu EZ-S machine, Shimadzu, Kyoto, Japan), and the debonding force was measured at the same time, as shown in Figure 5 and Figure 6, respectively. The maximum applied force is about 0.5 N, as there is no adhesion layer such as Ti or Cr. After metal transfer, metal film wrinkling patterns have been established on the Scotch tape due to a mechanical property mismatch between the two materials. 

**Figure 7:**
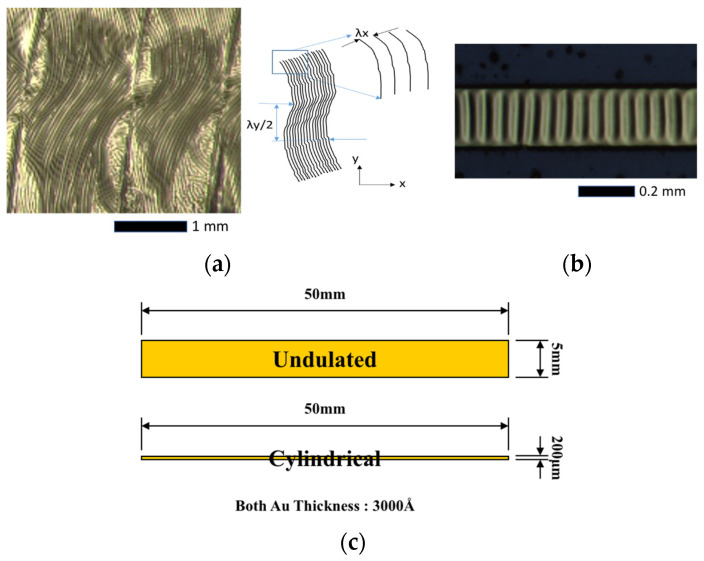
Wrinkling patterns of thin metal film are transferred onto Scotch tape, depending on the design dimensions. (**a**) Undulated wrinkling pattern; (**b**) Cylindrical wrinkling pattern; (**c**) dimension of thin metal film for undulated and cylindrical.

Figure 7 shows the undulated wrinkling pattern and the cylindrical one transferred to the Scotch tape. The lines between the undulated wrinkles are created by tape tests due to the angled tape attached to the metal pattern. The wrinkling patterns have been characterized with an optical microscope and SEM (scanning electron microscopy) in order to measure the wrinkling wavelength. The average values for the wavelengths in the direction of *x* and *y* are 57.8 μm (standard deviation of 0.62 μm) and 1115 μm (standard deviation of 78 μm), respectively. It should be noted that the wavelength of the cylindrical wrinkling, shown in Figure 7b, was measured at 55 µm in the previous work. The ratio of wavelengths in the *x* and *y* directions, L_*x*_/L_*y*_, is given by 0.052, which means the wrinkling in the *y* direction occurs just after the critical strain because it is close to *q* = 0. 

## 4. Three-Dimensional Direct FEM Modeling, Simulation and Results

**Figure 8:**
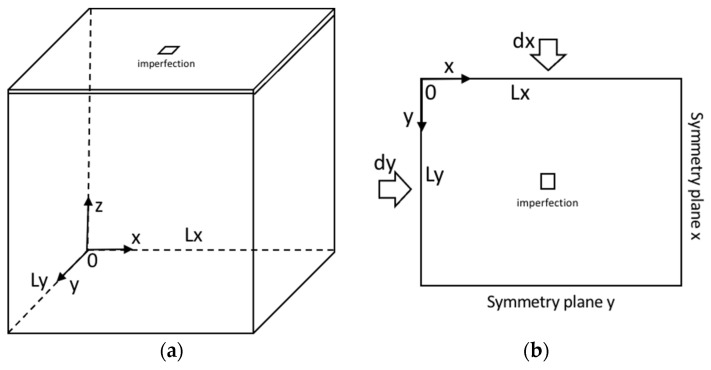
Three-dimensional wrinkling model and boundary conditions. (**a**) Overall view; (**b**) Top view.

A model consisting of a thin film bonded to a compliant substrate has been built with an embedded imperfection for 3D direct buckling simulation, as shown in Figure 8. It shows the boundary conditions and the applied displacements (or strains). The film surface is basically square or rectangular in shape depending on the loading conditions, such as equibiaxial or biaxial stressing, and the substrate was sufficiently thick in comparison with the film. 

The thin-film thickness is defined as 0.5 μm. Both the film and substrate materials are taken to be isotropic linear-elastic. Material properties needed for thin film and compliant substrates are Young’s moduli and Poisson ratios. The compliant substrate has 6.9 MPa and 0.45, while the thin film has 1 GPa and 0.42, respectively. One embedded imperfection, defined by the elastic property of the thin-film material instead of that of the substrate, is placed in the substrate adjacent to the film-substrate interface. To preserve symmetry, the imperfection with the square shape is put at the center of the plane. The dimension of the model has been chosen to be sufficiently large for the formation of different wrinkling patterns, such as cylindrical and undulated, as explained in the previous section. The boundary conditions U_*x*_ = 0, U_*y*_ = U_*z*_ = Free at *x* = L_*x*_, U_*y*_ = 0, and U_*x*_ = U_*z*_ = Free at *y* = L_*y*_ have been defined as symmetric planes, respectively. The vertex at the center in the bottom plane, *z* = 0, is regarded as fixed. Displacements at *x* = 0 and *y* = 0 are applied for the external loads. As a quarter model is used for the simulation, the results have been extended into a full model. 

Biaxial surface wrinkling has been simulated by applying transversal strain (*d_x_*) and longitudinal strain (*d_y_*) at the same time, and thus, biaxiality ratio (BR), defined as Equation (9), is used as a parameter of simulation with transversal strain. Simulations for various biaxiality ratios were performed in such that *d_x_* was kept constant and *d_y_* was varied within the range of 0 ≤ *d_y_* ≤ *d_x_* for different cases of BR. As a consequence, the biaxiality varies within the range of 0 ≤ BR ≤ 1, where BR = 0 assimilates with uniaxial compression (applied in the *x*-direction) and BR = 1 corresponds to perfectly equibiaxial compression.

(11) $$\mathrm{Biaxiality}\;\mathrm{Ratio}\;(\mathrm{BR})=\frac{d_{y}}{d_{x}}$$

**Figure 9:**
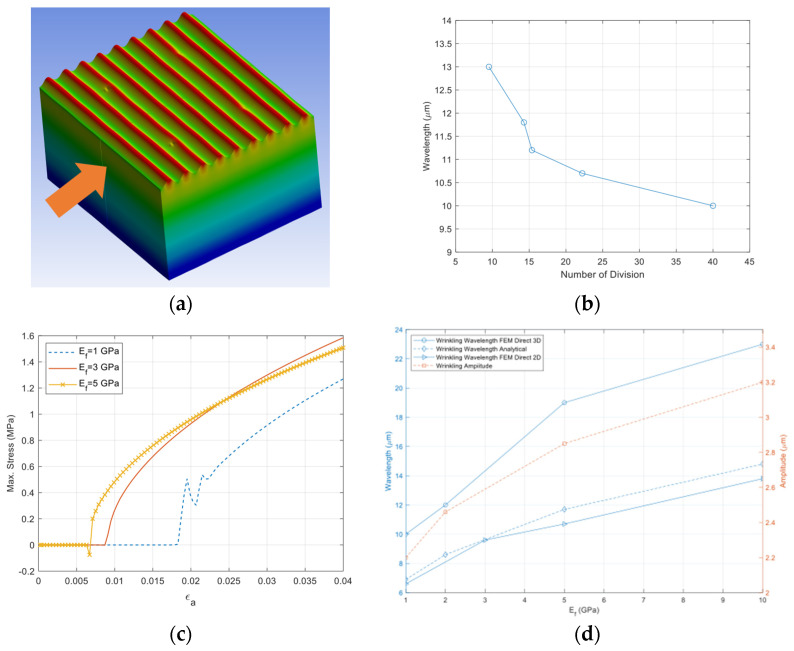
Cylindrical wrinkling and its wrinkling characteristics. (**a**) Cylindrical wrinkling; (**b**) Wrinkling wavelength vs. mesh size; (**c**) Critical strain vs. *E_f_*; (**d**) Wrinkling parameters vs. *E_f_*.

Verification of the model has been performed with the case of uniaxial compression (BR = 0), as it is considered the initial wrinkling state for the following complex wrinkling patterns. The wavelength variation resulting from the simulation depending on the mesh size has been first investigated, and then wrinkling behaviors have been simulated. Figure 9a shows the cylindrical wrinkling pattern due to the uniaxial straining and its wrinkling characteristics. The wavelength has little dependency on the mesh size, as presented in Figure 9b. It is noteworthy that finer meshes provide a more stable wavelength value. By referring to maximum stress as a function of applied strain (*ε_a_*), critical strains initiating the cylindrical wrinkling can be found, as shown in Figure 9c. Figure 9d presents wrinkling parameters in terms of wavelength and amplitude, which can be used for material property extraction of the thin film. Note that the wrinkling wavelength from 3D direct simulation is greater than that of 2D direct and analytical solutions. As the elasticity of the thin film (*E_f_*) increases, the critical strain becomes smaller.

**Figure 10:**
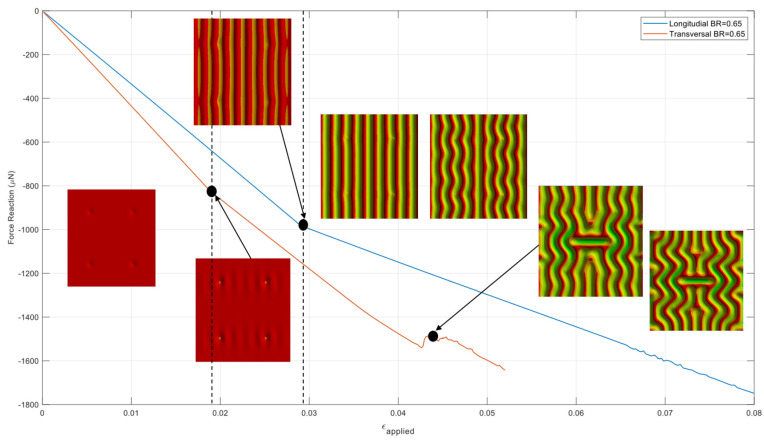
Force reaction at the loading surfaces when BR is 0.65.

Before the wrinkling behavior study of the model with a full range of biaxial loading, the wrinkling pattern change was investigated in the case of BR = 0.65. Figure 10 shows force reactions on the surfaces where longitudinal and transversal displacement loading are applied as a function of the applied strain. The entire wrinkling pattern, from the pre-instability stage to the final stage, has been captured n one simulation run. The reaction force curves introduce sudden changes in slope or load drop whenever the wrinkling mode change occurs. Starting from the flat surface of the thin film before the critical strain, slight thin-film wrinkling has occurred at the critical strain around imperfection sites. The onset of the initial wrinkling is at the applied strain of approximately 1.8 × 10^−2^. The cylindrical wrinkling mode appeared at the applied strain of approximately 2.8 × 10^−2^ and lasted through the strain of approximately 3.2 × 10^−2^ before the onset of the undulated (or herringbone) mode. From a strain of approximately 4.4 × 10^−2^, the wrinkling pattern has been changed to labyrinth mode. Depending on the wrinkling mode, a longitudinal or transversal force reaction can be used to refer to the wrinkling mode change.

**Figure 11:**
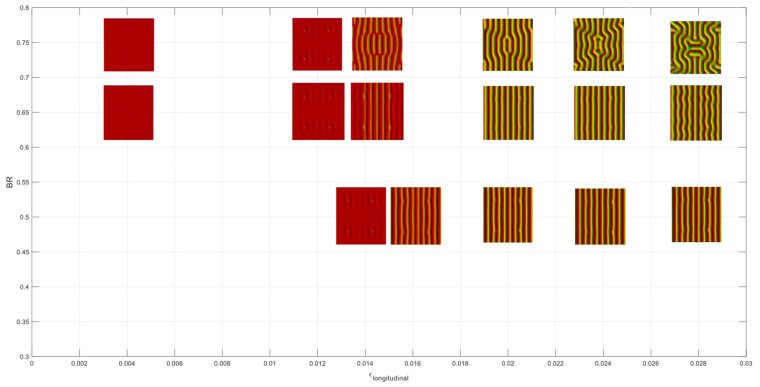
Wrinkling patterns for biaxiality ratios between 0.3 and 0.8.

To assess the dependency of wrinkle patterns on the biaxiality ratio, extensive simulations have been performed by monitoring the pattern evolution. The resulting wrinkling patterns are presented in two parts, as a more detailed examination is required in the cases of BR between 0.9 and 1.0 due to the nature of thin-film buckling. Figure 11 shows the top view of the wrinkling patterns for biaxiality ratios between 0.3 and 0.8. It is found that cylindrical wrinkling is the dominant mode until a biaxiality ratio of 0.5 in the range of the longitudinal strain of interest. In the case of BR = 0.65, 1D cylindrical wrinkling has been changed to a slightly undulated model from 2.8 × 10^−2^ strain input. The 1D cylindrical wrinkling has disappeared from 0.75 of BR as the undulated mode is dominant after the critical strain, and it becomes labyrinth mode at the highest strain input. The color contours represent the out-of-plane displacement, with the red and yellow (or orange) colors being the highest (peak) and lowest (valley) positions, respectively.

**Figure 12:**
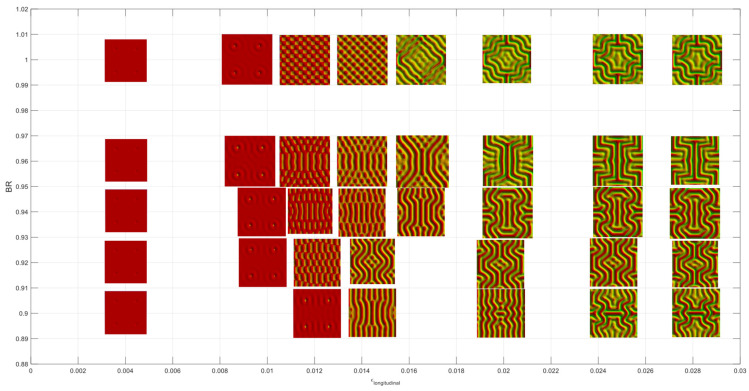
Wrinkling patterns for biaxiality ratios between 0.9 and 1.

The evolution of the wrinkle patterns for BR values greater than 0.9 is shown in Figure 12. It can be seen that 1D cylindrical wrinkling dominant in the BRs lower than 0.9 is suppressed, and undulated or checkerboard-like modes appear just after critical stains. Note that the critical strain value becomes slightly smaller from the BR of 0.92, and initial wrinkling patterns tend to be circular near the imperfection sites with the BR value greater. These primary modes change more complex patterns or more continuous wrinkles, such as various labyrinth structures, as the applied strain increases.

**Figure 13:**
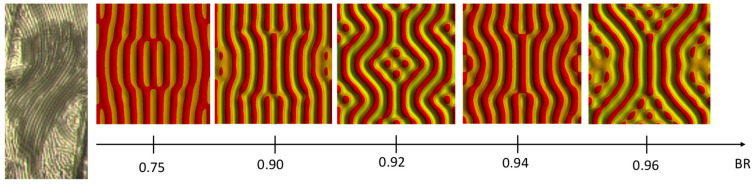
Comparison between experiment and undulated simulation patterns of different BRs.

Given various wrinkling patterns depending on the BR, undulated wrinkles close to the experimental result can be extracted, as shown in Figure 13. From the experiment, it is seen that the undulated wrinkle has two different regions: the undulated pattern itself and the arbitrary pattern. The arbitrary pattern is near the boundary line created due to the angle between the tape and the wafer during the tape test. The closest pattern to the experiment in view of the undulated one and the arbitrary one is the wrinkling pattern with BR 0.92, as it clearly shows two different zones due to the boundary conditions. It is also confirmed that the wavelength of the longitudinal direction is much shorter than that of the transversal one, as are the experimental results. The simulation result with a BR of 0.92 gives wavelengths in longitudinal and transversal directions of 10 μm and 50 μm, respectively. Thus, the q value is estimated at 0.2, which is much greater than the experimental one, 0.052. 

## 5. Conclusions and Perspectives

Thin-film wrinkling due to a mechanical mismatch between elastic thin metal film and a compliant substrate is frequently encountered in microfabrication. A biaxial wrinkling pattern of a thin film has been fabricated with the transfer of thin metal film into Scotch tape by adjusting the width of the thin film from our previous cylindrical wrinkling metal pattern design. To understand the experimental pattern, 3D direct FEM modeling and simulation based on the embedded imperfection approach have been carried out. The technique is easily implemented for structures consisting of thin films above a compliant substrate without treating the surface patterns discretely under specific assumptions. The generation of wrinkling morphologies and their transformations have been directly obtained from the simulations. The established model has successfully produced the cylindrical, 1D wrinkling pattern with one directional strain input. The biaxial wrinkling has been performed with a parameter defined as BR (biaxiality ratio). It has been found that the 1D wrinkling pattern is dominant when BR approaches 0.9, while the undulated pattern appears at 0.9 of BR. Given that the BR is larger than 0.9, the wrinkling patterns change to cylindrical mode, undulated mode, or checkerboard mode for equibiaxial straining and labyrinth mode. From the undulated patterns depending on the BR, the wrinkling pattern closest to the experiment has been found to be able to estimate the applied strain. It is worth noting that all the results presented here are based on simple isotropic linear elastic material behavior with an initially perfectly flat film layer. The 3D direct model and simulation should be simple and robust for future investigations involving more complex material, geometric, and loading conditions. 

## Data Availability

The original contributions presented in the study are included in the article, further inquiries can be directed to the corresponding author.

## References

[1] Wang S., Song J., Kim D.H., Huang Y., Rogers J.A. (2008). Local versus global buckling of thin films on elastomeric substrates. Appl. Phys. Lett..

[2] Ma Y., Xue Y., Jang K.I., Feng X., Rogers J.A., Huang Y. (2016). Wrinkling of a stiff thin film bonded to a pre-strained, compliant substrate with finite thickness. Proc. R. Soc. A Math. Phys. Eng. Sci..

[3] Breid D., Crosby A.J. (2009). Surface wrinkling behavior of finite circular plates. Soft Matter.

[4] Barth J.V., Costantini G., Kern K. (2005). Engineering atomic and molecular nanostructures at surfaces. Nature.

[5] Yoo P.J., Suh K.Y., Kang H., Lee H.H. (2004). Polymer elasticity-driven wrinkling and coarsening in high temperature buckling of metal-capped polymer thin films. Phys. Rev. Lett..

[6] Bowden N., Huck W.T.S., Paul K.E., Whitesides G.M. (1999). The controlled formation of ordered, sinusoidal structures by plasma oxidation of an elastomeric polymer. Appl. Phys. Lett..

[7] Bowden N.B., Brittain S.T., Evans A.G., Hutchinson J.W., Whitesides G.M. (1998). Spontaneous Formation of Ordered Structures in Thin Films of Metals Supported on an Elastomeric Polymer. Nature.

[8] Yoo P.J., Suh K.Y., Park S.Y., Lee H.H. (2002). Physical Self-Assembly of Microstructures by Anisotropic Buckling. Adv. Mater.

[9] Chen X., Hutchinson J.W. (2004). Herringbone buckling patterns of compressed thin films on compliant substrates. J. Appl. Mech..

[10] Chung J.Y., Nolte A.J., Stafford C.M. (2011). Surface Wrinkling: A Versatile Platform for Measuring Thin-Film Properties. Adv. Mater..

[11] Seok S., Park H.D., Kim J. (2022). Scotch-tape surface wrinkling based thin-film material properties extraction. J. Micromech. Microeng..

[12] Song J., Jiang H., Choi W.M., Khang D.Y., Huang Y., Rogers J.A. (2008). An analytical study of two-dimensional buckling of thin films on compliant substrates. J. Appl. Phys..

[13] Stafford C.M., Harrison C., Beers K.L., Karim A., Amis E.J., VanLandingham M.R., Kim H.C., Volksen W., Miller R.D., Simonyi E.E. (2004). A buckling-based metrology for measuring the elastic moduli of polymeric thin films. Nat. Mater..

[14] Stafford C.M., Harrison C., Karim A., Amis E.J. (2002). Measuring the modulus of polymer films by strain-induced buckling instabilities. Polymer.

[15] Yin J., Chen X., Sheinman I. (2009). Anisotropic buckling patterns in spheroidal film/substrate systems and their implications in some natural and biological systems. J. Mech. Phys. Solids.

[16] Huang Z., Hong W., Suo Z. (2004). Evolution of wrinkles in hard films on soft substrates. Phys. Rev. E.

[17] Yin J., Chen X. (2010). Elastic buckling of gradient thin films on compliant substrates. Phil. Mag. Lett..

[18] Huang Q., Xu R., Liu Y., Hu H., Giunta G., Belouettar S., Potier-Ferry M. (2017). A two-dimensional Fourier-series finite element for wrinkling analysis of thin films on compliant substrates. Thin-Walled Struct..

[19] Mei H., Landis C.M., Huang R. (2011). Concomitant wrinkling and buckle-delamination of elastic thin films on compliant substrates. Mech. Mater..

[20] Leifer J., Belvin W. Prediction of wrinkle amplitudes in thin film membranes using finite element modeling. Proceedings of the 44th AIAA/ASME/ASCE/AHS/ASC Structures, Structural Dynamics, and Materials Conference.

[21] Siavash N., Ryu D., Shen Y.-L. (2019). Direct numerical simulation of buckling instability of thin films on a compliant substrate. Adv. Mech. Eng..

[22] Siavash N., Ryu D., Shen Y.-L. (2020). Instabilities of thin films on a compliant substrate: Direct numerical simulations from surface wrinkling to global buckling. Sci. Rep..

[23] Nikravesh S., Ryu D., Shen Y.-L. (2022). Surface Wrinkling versus Global Buckling Instabilities in Thin Film-Substrate Systems under Biaxial Loading: Direct 3D Numerical Simulations. Adv. Theory Simul..

[24] Seok S., Park H., Coste P., Kim J. (2023). Direct Numerical Simulation of Surface Wrinkling for Extraction of Thin Metal Film Material Propertie. Micromachines.

[25] Audoly B., Boudaoud A. (2008). Buckling of a stiff film bound to a compliant substrate—Part I Formulation, linear stability of cylindrical patterns, secondary bifurcations. J. Mech. Phys. Solids.

